# Trends in Semen Quality: A Contrasting Perspective From a Single‐Centre Review in Ireland

**DOI:** 10.1111/andr.70193

**Published:** 2026-02-12

**Authors:** Ciara Nolan, Hayley Jackson, Aisling Looney, Jennifer Cullinane, Louise E. Glover, David Crosby, Cathy Allen

**Affiliations:** ^1^ Merrion Fertility Clinic Dublin Ireland; ^2^ National Maternity Hospital Dublin Ireland; ^3^ School of Medicine University College Dublin Dublin Ireland; ^4^ St. Vincent's University Hospital Dublin Ireland

**Keywords:** fertility rates, male fertility, male health, microplastics, sperm concentration

## Abstract

**Introduction:**

Male factor contributes up to half of infertility cases and reports of a global decline in semen quality raise concern for the future of male reproductive health. However, fertility trends can vary by region and population. Interpreting local patterns is crucial for understanding the true trajectory of male fertility. We describe longitudinal trends in semen parameters among men attending a single fertility clinic in Ireland.

**Methods:**

We retrospectively analysed 18,219 semen analyses for 15,413 men attending a fertility clinic between 2008 and 2023. Sperm concentration, progressive motility and total motile sperm count (TMSC) were analysed, with 2021 WHO criteria applied to identify subnormal results. Non‐parametric tests were applied to assess temporal changes (Kruskal–Wallis with Dunn's post‐hoc test for multiple comparisons). A longitudinal subanalysis compared first and last semen samples among men with repeat testing, and grouped by time interval (<1 year, 1–3 years, 3–5 years, >5 years).

**Results:**

Median sperm concentration increased significantly from 57 M/mL in 2008 to 70 M/mL in 2023 (*p* < 0.0001), representing a 22.8% rise. TMSC showed a slight, non‐significant rise, while progressive motility remained stable over time. The proportion of oligospermic (<16 M/mL) and azoospermic (0 M/mL) samples was unchanged between 2008 (19.5% and 3.7%) and 2023 (21% and 3.6%). Longitudinal intra‐individual analysis demonstrated no significant changes in semen parameters across short, medium and long‐term follow‐up, except for reduced semen volume at 3–5 years.

**Conclusions:**

Contrary to concerning reports of a global decline in semen quality, we observed a significant increase in sperm concentration over a 16‐year period, with stable rates of oligospermia and azoospermia. These findings emphasise the importance of regional differences and suggest that local factors such as healthcare access and lifestyle may play a role. Examining local trends provides a more nuanced understanding of overall male fertility trends and may inform targeted clinical and public health strategies.

## 1 Introduction

Over the past few decades, numerous studies have reported a concerning decline in semen quality and male fertility rates globally [1, 2, 3, 4], such that male factor is now a contributory cause in up to half of infertility cases [5]. A 2017 meta‐analysis of men unselected by fertility status showed that across North America, Europe and Australia, sperm concentrations declined by 52.4% (−1.4% per year) and total sperm counts declined by 59.3% (−1.6% per year) over a 72‐year period. The findings demonstrated no sign of a “levelling off” in the decline, and more recent reports show a similar trend across South America, Africa and Asia [1, 2]. Population‐level estimates suggest that as many as 7%–10% of all men have at least one semen parameter that falls below the World Health Organisation's (WHO) reference thresholds, even affecting young men (<30 years) [6, 7, 8, 9].

The criteria for “normal” semen parameters have evolved over the years to reflect changes in male fertility rates. The WHO reference values are derived from the lowest fifth percentile of results from men who have contributed to a natural conception within 12 months of trying. This value has changed from 60 million sperm/mL (M/mL) in the 1940s [10, 11] to 20 M/mL in the 1980s [12], with a further decrease to 16 M/mL in 2021 [13, 14].

While these reports raise concern about the future of human reproduction and the impact on fertility rates, the landscape of semen quality trends is not consistent. A 2023 meta‐analysis on both fertile men and men unselected by fertility status in America found no clinically significant decline in sperm concentration. Some studies included in the report even showed an increase between 1970 and 2018 [15]. Similarly, several large multinational reviews rebut a uniform decline in global semen quality [16, 17, 18, 19, 20], highlighting that the overall trajectory remains uncertain and male fertility trends continue to be debated [21, 22].

Heterogeneity in populations, laboratory methods and confounders may strongly influence sperm fertility trends, highlighting the importance of interpreting data from different geographical, demographic and healthcare systems [22]. While some of the factors attributed to the decline in male fertility are reversible and may be improved with attention to diet, lifestyle and body mass index (BMI), there is a growing concern that factors such as pollution, microplastics and environmental toxins are contributing to the decline [23, 24, 25, 26, 27, 28, 29].

Moreover, semen quality is seen as a marker of overall male health [30, 31], and thus describing temporal trends in sperm parameters may have implications far beyond fertility and subfecundity. Understanding local trends in sperm parameters is crucial for providing targeted interventions and counselling to improve reproductive outcomes and to guide further research.

This study aims to investigate the longitudinal trends in sperm parameters among men attending a single fertility clinic in Ireland. This will allow comparison of findings with international data and facilitate a deeper reflection on overall male health and potential local contributory causes to infertility.

## 2 Methods

### 2.1 Study Design

We conducted a retrospective analysis of all adult semen analyses performed between the period of January 2008 and December 2023 (16‐year period) at Merrion Fertility Clinic, a not‐for‐profit fertility clinic performing over 1000 cycles of in vitro fertilisation (IVF) per year. In accordance with ethical standards, this study was performed in compliance with the Declaration of Helsinki. Ethical approval was waived, as the analysis of fully anonymised retrospective data does not require approval under our institution's guidelines. Similarly, individual patient consent was not required.

### 2.2 Patient Population

Semen and sperm parameters for all adult (>18 years) male patients who attended the fertility clinic were included in the analysis, regardless of clinical indication. Male patients with active malignancy were not managed at our clinic and were therefore excluded. Semen analyses performed for social sperm banking purposes (including a small number of transgender patients) or for pre‐vasectomy banking were included. Some men provided repeat samples within the same calendar year; to avoid intra‐individual clustering and to capture population estimates of semen quality, the first sample from each man within the calendar year was included for longitudinal data analysis. This approach (single sample per man per year) has been deemed adequate for assessing epidemiological differences in semen quality across populations [32, 33, 34, 35]. Among the men who provided more than one semen sample during the study period, a sub‐analysis was performed to assess within‐individual changes in parameters over time. For these men, the first and last samples were included to minimise intra‐individual variability while capturing longitudinal change.

### 2.3 Semen Analysis

Semen samples were collected after 2–5 days of sexual abstinence, in sterile plastic containers (Sarstedt, Numbrecht, Germany) and allowed to liquefy for 30 min at 37°C. Semen analysis was performed by trained embryologists within one hour of ejaculation according to the World Health Organisation (WHO) recommendations [13]. Sperm concentration was determined using a Makler counting chamber. In cases where results were borderline normal, a haemocytometer was used to confirm accuracy. Computer Assisted Sperm Analysis (CASA) was not used at any point during the study period. Primary outcome variables for each ejaculate were sperm concentration (million/mL; 10^6^/mL), progressive motility (percentage of spermatozoa moving with forward progression [A + B]), and total motile sperm count (TMSC) (semen volume (mL) × sperm concentration (M/mL) × proportion of progressively motile spermatozoa). While most samples were processed according to the 2010 WHO criteria in place at the time of collection [36], for the purpose of data analysis, the most recent 2021 WHO criteria were applied retrospectively to identify abnormal results (concentration <16 M/mL) [13]. The laboratory undergoes annual External Quality Control testing to maintain accurate and reliable results and to ensure consistent adherence to WHO protocols.

### 2.4 Statistical Analysis

Statistical analysis was performed using GraphPad Prism (GraphPad Software Inc., San Diego, CA). Shapiro–Wilk and Kolmogorov–Smirnov tests were used to test for normality and showed non‐parametric variables. Kruskal–Wallis with Dunn's post‐hoc test for multiple comparisons was used as appropriate to assess changes in median age, sperm concentration, motility and TMSC over time. For categorical variables (normal, oligospermic and severely oligospermic classifications), χ^2^ test was used; *p* < 0.05 was considered significant.

To perform a longitudinal sub‐analysis of men with repeat samples, men were stratified into four groups according to the time interval between their first and last semen analysis: <1 year, 1–3 years, 3–5 years, >5 years. Semen analyses repeated <3 months apart were excluded. This approach has been previously used to observe short, medium and long‐term changes in parameters [37]. Given the non‐parametric distribution of parameters, paired non‐parametric testing was performed using the Wilcoxon signed rank test to compare the first and last semen samples. Statistical significance was defined as *p* < 0.05.

## 3 Results

Sperm parameters were analysed for 15,413 male patients who underwent a total of 18,219 semen analyses during the 16‐year study period. Patient demographics and sperm parameter data are summarised in Table 1.

**TABLE 1:** Patient age and sperm parameter data from 2008 to 2023.

Parameters	2008	2009	2010	2011	2012	2013	2014	2015	2016	2017	2018	2019	2020	2021	2022	2023	*p* value
Semen analyses (*n*)	926	984	1106	1118	1053	1202	1245	1241	1186	1114	1071	1121	1027	1405	1178	1242	
Patients (*n*)	752	769	890	873	894	1029	1069	1096	1039	955	952	926	895	1184	1027	1063	
Age (years)	36	36	36	36	36	36	36	36 ^a^	36 ^a^	37 ^a^	37 ^a^	37 ^a^	37 ^a^	38 ^a^	37 ^a^	37 ^a^	<0.0001
	(33–39)	(33–39)	(33–39)	(33–39)	(33–39)	(33–40)	(33–39)	(33–40)	(33–40)	(34–40)	(34–40)	(34–40)	(34–40)	(34–41)	(34–40)	(34–40)	
Sperm concentration (x10^6^/mL)	57	59	58	44^a^	56	55	46 ^a^	50	50	46 ^a^	48 ^a^	55	60	60	72 ^a^	70 ^a^	<0.0001
	(20–98)	(25–92)	(26–89)	(16–80)	(22–95)	(23–94)	(21–78)	(21–89)	(23–87)	(18–81)	(23–81)	(22–90)	(22–89)	(24–90)	(23–108)	(24–110)	
Progressive motility (grade A + B; %)	55	55	55	53	49 ^a^	50 ^a^	51	57 ^a^	56	55	51	59 ^a^	52	54	55	54	<0.0001
	(40–65)	(40–66)	(40–67)	(36–65)	(34–63)	(35–62)	(37–62)	(43–68)	(42–66)	(39–68)	(40–63)	(272)	(37–66)	(38–66)	(42–67)	(42–64)	
TMSC (x10^6^ per ejaculate)	86	81	85	62 ^a^	74	77	68 ^a^	82	81	68	70 ^a^	89	77	81	99	103	<0.0001
	(25–181)	(27–159)	(24–176)	(15–139)	(22–166)	(23–149)	(22–137)	(28–166)	(28–157)	(20–154)	(27–133)	(26–169)	(21–156)	(25–161)	(30–189)	(25–195)	

*Note*: Results are depicted as median values with interquartile range (IQR) in parentheses. Comparison of parameters across years was performed using Kruskal‐Wallis test for non‐parametric data.

Abbreviation: TMSC, total motile sperm count.

^a^ *p* <0.05 vs 2008, Dunn's post‐hoc test.

Median age of men attending the clinic for investigation increased over the study period, with significant differences noted between 2008 and 2015 onwards. In 2008, the median male age was 36 years (range 20–64), as compared to 37 years (range 18–62) in 2023. The number of semen analysis tests performed in the clinic increased by 37.8% over the included time frame, from 926 tests in 2008 to 1242 performed in 2023 (Table 1).

**FIGURE 1:**
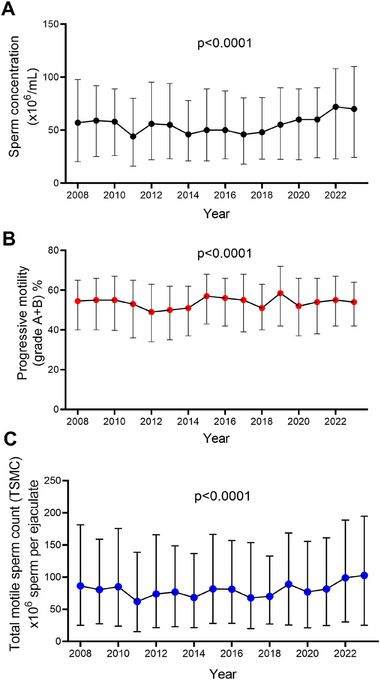
Changes in sperm parameters from 2008 to 2023. Data is represented as median and interquartile range, *p* < 0.0001 (Kruskal–Wallis). (A) Sperm concentration (10^6^/mL); (B) progressive motility (%); (B) total motile sperm count (10^6^ motile sperm per ejaculate).

Sperm concentration trends indicated an overall increase between 2008 and 2023. Median sperm concentration increased from 57 M/mL (range 0–340 M/mL) in 2008 to 70 M/mL (range 0–270 M/mL) in 2023 (*p* < 0.0001, Kruskal–Wallis), representing a rise of 22.8% in this 16‐year period. A largely upward trend in median sperm concentration is noted from 2020 onwards (Figure 1A). While progressive sperm motility (%) trends did not indicate a similar upward trajectory (Figure 1B), median total motile sperm count (TMSC) showed a slight increase between 2008 (86.3 × 10^6^ spermatozoa, range 0–961 × 10^6^) and 2023 (102.7 × 10^6^ spermatozoa, range 0–1246 × 10^6^), as outlined in Figure 1C, however, this was not significant.

**FIGURE 2:**
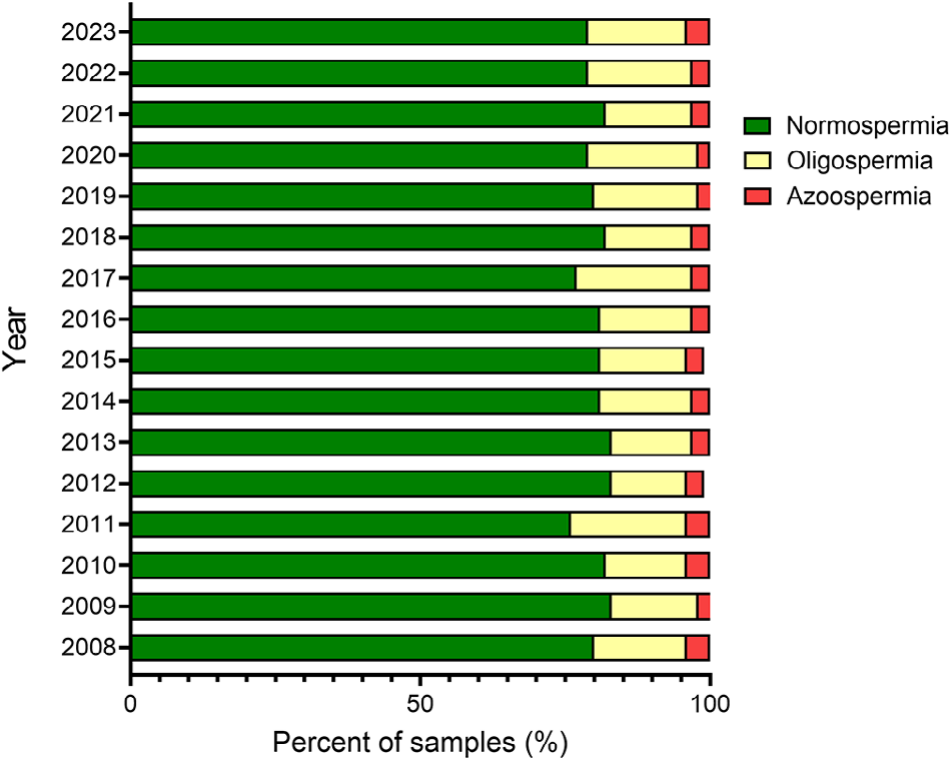
Proportion of semen analysis samples from 2008 to 2023 with normospermia, oligospermia, and azoospermia.

Sperm concentration test results were further stratified into normal, oligospermia and azoospermia to investigate annual trends of subnormal sperm concentrations over the study period. The proportion of oligospermic samples, that is, sperm concentration of less than 16 M/mL, as defined by the WHO [13], was not significantly changed from 2008 (19.5%) to 2023 (21%) (Figure 2) in the 15,413 samples analysed. The proportion of semen analyses that showed azoospermia (no spermatozoa seen in the sample) was similarly unchanged from 2008 to 2023 (3.7% and 3.6%, respectively; Figure 2).

**FIGURE 3:**
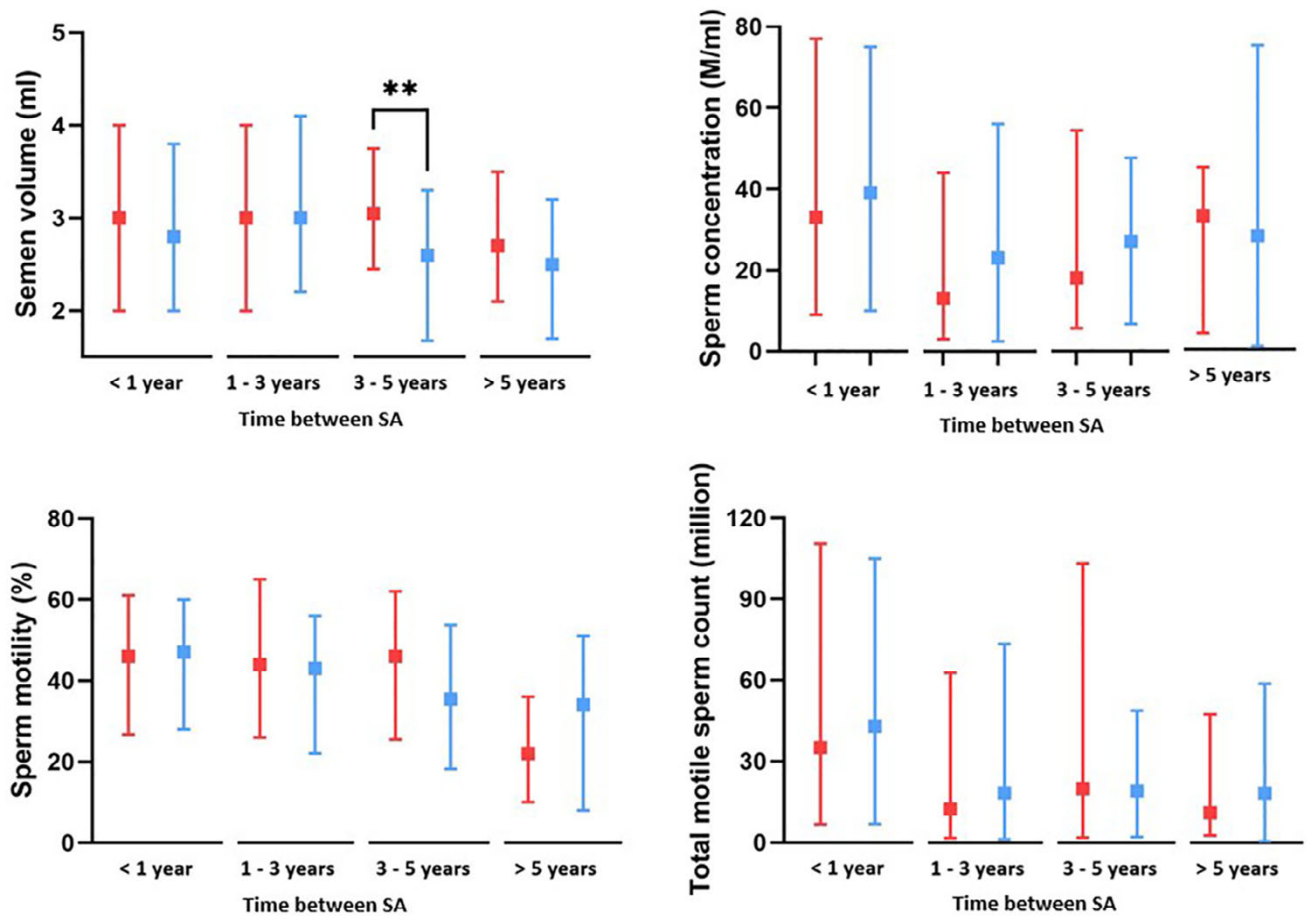
Intra‐individual changes in sperm parameters among men providing repeat samples. Median and interquartile range for each sperm parameter are illustrated, with paired comparisons between first and last semen sample analysed within 1 year (*n* = 919), between 1 and 3 years (*n* = 79), between 3 and 5 years (*n* = 28), and over 5 years (*n* = 11). **p〈0.01, using the Wilcoxon matched pairs signed rank test.

A subset of men attended the clinic on more than one occasion and provided repeat semen analysis samples. Comparison of the first and last sample for each individual revealed no statistically significant differences in sperm concentration, progressive motility, or TMSC across any of the four timeframes (< 1 year, 1–3 years, 3–5 years, >5 years). A statistically significant change in semen volume was observed when the sample was repeated within a 3–5 year time frame; no significant differences in volume were observed within other timeframe groups. The results of the longitudinal sub‐analysis are illustrated in Figure 3.

## 4 Discussion

The observed increase in sperm concentration of nearly 23% over the study period is unexpected and contrasts with widely cited reports of a global decline in sperm quality. These findings add to a growing body of literature that challenges the concern of a widespread detrimental decline in semen quality and highlight the importance of regional, population‐specific data when interpreting temporal trends.

The significant increase in median sperm concentration within this cohort stands in contrast with the landmark 2017 meta‐analysis reporting >50% decline in sperm concentration among men unselected by fertility status across Western countries [1]. However, more recent studies have questioned the generalisability of these findings, given the considerable heterogeneity among study populations, laboratory techniques and abstinence times [15, 16, 17, 18, 19, 20, 21, 22]. Importantly, a 2023 meta‐analysis of men in North America showed no meaningful decline in sperm concentration, with some included studies even demonstrating an increase over time [15]. Our findings are concordant with this more recent evidence and support the picture that trends in semen quality may not be globally uniform.

It is important to interpret the findings in context, and several factors may account for the disparity observed. Ireland has experienced substantial public health improvements over the past two decades, leading to reductions in smoking prevalence [38], declines in alcohol consumption [39], and an increased awareness of healthy lifestyle choices [40]. Men with better overall health have been shown to have improved semen parameters [30, 31, 41, 42]. This shift towards healthier living, combined with increased health literacy and engagement in preventative healthcare, may have contributed to the increase in sperm concentration seen.

Notably, the observed increase in sperm concentration occurred despite a small but significant rise in paternal age. Advancing paternal age is typically associated with reductions in semen volume, concentration, motility and DNA integrity [43, 44]. However, the improvement in sperm concentration seen here, in an ageing cohort, may suggest true population‐level improvements that offset any expected age‐related decline.

Interestingly, while sperm concentration increased over time, progressive motility and total motile sperm count (TMSC) did not show a similar trajectory. These divergent trends highlight the limitation of focusing on sperm concentration alone when assessing male fertility patterns. While sperm concentration has previously been favoured for epidemiological studies [45], TMSC is increasingly recognised as a more clinically relevant predictor of reproductive outcomes, particularly in the setting of natural fertility and intrauterine insemination [46, 47]. The relative stability in TMSC in our cohort suggests that improvements in concentration alone may not necessarily translate into enhanced overall fertility potential, and underscores the multifactorial nature of male fertility.

The number of semen analyses performed in our clinic increased by 37.8% during the 16‐year study period. This may be explained by the expansion of fertility clinics and services in Ireland, making fertility testing more accessible. Media coverage and celebrity endorsements of fertility treatments have reduced the stigma associated with fertility testing, while the inclusion of fertility treatments in some healthcare insurance policies has also made it more affordable. However, despite both the increase in testing and the increase in median sperm concentration over time, the proportion of oligospermic (<16 M/mL) and azoospermic (0 M/mL) results has remained stable over the same timeframe. This divergence may reflect different etiologies and factors influencing semen quality. An overall improvement in sperm concentration may be related to modifiable factors such as lifestyle, increased health awareness and earlier engagement with fertility services. In contrast, the proportion of men with frankly impaired sperm concentration may be caused by persistent and less modifiable factors. The stable rates of oligospermia and azoospermia may reflect etiologies such as genetic, endocrine or testicular factors that are unlikely to change with lifestyle measures. This observation may suggest that public health interventions and proactive health behaviours can benefit men with mild to moderate reductions in semen quality, while the burden of severe impairments remains unchanged.

In contrast to the population‐level observation of a rise in sperm concentration over time, a longitudinal sub‐analysis of men who provided repeat semen samples demonstrated no significant change in parameters over short, medium or long term follow‐up intervals. The only exception was a statistically significant decrease in semen volume in men with a 3–5 year interval between samples. However, this isolated finding was not accompanied by changes in sperm concentration, motility or TMSC, suggesting limited clinical relevance. Semen volume is known to be particularly sensitive to factors such as abstinence duration, accessory gland function and hormonal fluctuations, and thus may exhibit greater intra‐individual variability than other sperm parameters [48, 49]. The absence of consistent volume changes over time and the lack of associated changes in TMSC suggest that this finding may reflect physiological variability, rather than meaningful impacts on fertility potential.

While this study provides valuable insights into semen trends at our clinic, several limitations must be acknowledged. First, there is a lack of detailed demographic and clinical information recorded for the men included in the study. Without comprehensive data on factors such as socioeconomic status, BMI, smoking status, lifestyle habits, and underlying health conditions, it is challenging to account for confounding factors. The lack of detailed information on the men may also reflect a societal impression that male factor infertility is less contributory to fertility problems, potentially leading to a gap in the collection of comprehensive male demographic data.

The increase in the number of semen analysis tests performed over the study period raises the possibility of sampling bias, particularly if health‐conscious, proactive men have accessed testing in recent years. Men with greater health literacy are more likely to follow healthier lifestyle practices and are more likely to have normal semen parameters as a result [30, 41], potentially skewing the trend towards an apparent increase in sperm concentration. However, as socioeconomic and lifestyle data were not available, we cannot directly evaluate its influence on our cohort. Importantly, the proportion of abnormal results remained consistent across the years, suggesting that even if such a sampling bias were present, it did not dilute the representation of men with impaired semen quality.

The study population is relatively homogeneous, consisting primarily of men who accessed private healthcare and self‐funded fertility treatments. This may limit the generalisability of our findings. It is noteworthy that publicly funded fertility treatments were introduced in Ireland after the study period (September 2023), which is expected to diversify the population accessing the clinic and testing indications, providing an important opportunity for future evaluation of semen quality trends in a broader demographic.

Finally, a methodological explanation for our contrasting results must be considered. All semen analyses were performed in a single laboratory that uses consistent procedures according to WHO guidelines, and with regular participation in External Quality Control programmes. While minor procedural shifts such as equipment upgrades, changes in staff, or calibration adjustments over long timeframes cannot be entirely excluded, stringent Internal and External Quality Control measures are in place to maintain consistency of results and minimise the likelihood that a methodological drift explains the observed trends. While a small proportion of samples were collected off‐site, recent evidence suggests no difference in semen parameters between samples produced off‐site and those collected in‐clinic [50], and is therefore unlikely to contribute to the findings.

The single‐centre setting serves as a notable strength of the study. It ensures consistency in methodology and in the electronic reporting of patient records, reducing the variability that might arise from multicentre studies.

Additionally, despite a change in the lower reference value used for a “normal” semen analysis during the study period [13, 14], a consistent approach was used to retrospectively apply the most recent WHO criteria to identify abnormal results (concentration <16 M/mL) [13]. This uniform threshold enhances the comparability of data across time.

Further strengths of the study that enhance the robustness and relevance of the findings include the substantial sample size comprising 15,413 male patients and 18,219 semen analyses collected over a 16‐year period, allowing for the analysis of long‐term trends in semen parameters. The protracted timeframe helps to mitigate any short‐term fluctuations and provides a more comprehensive overview of longitudinal patterns.

Finally, the ability for men to self‐refer for fertility testing without prolonged delays may be particularly relevant to the interpretation of these findings. Earlier access to investigation may capture a more proactive subset of the population, where heightened health awareness and early lifestyle modifications occur before or alongside fertility testing. While our findings contrast with the narrative of a universal decline in male fertility, they highlight the importance of contextualising local data. Perhaps under certain conditions, such as motivated, health‐conscious populations with proactive behaviours, semen quality may remain stable or even show an improvement over time.

## 5 Conclusion

The observed increase in sperm concentration within our clinic stands in contrast to international trends, suggesting the presence of unique factors influencing male fertility within our patient population. This rise, observed over a 16‐year period, highlights the importance of regional data, healthcare context, and population characteristics when interpreting male fertility trends. Overall, this study helps develop a nuanced understanding of male reproductive health, highlighting that while global trends may point towards declining semen quality, localised differences are possible and warrant further investigation.

## Author Contributions

**Ciara Nolan**: conceptualisation, methodology, investigation, data curation, formal analysis, writing – original draft, review and editing. **Hayley Jackson**: methodology, data curation, writing – original draft, review and editing. **Aisling Looney**: supervision, writing – original draft, review and editing. **Jennifer Cullinane**: conceptualisation, methodology, investigation, data curation. **Louise E. Glover**: conceptualisation, methodology, investigation, data curation, formal analysis, writing – original draft, review and editing, supervision. **David Crosby**: conceptualisation, methodology, writing – review and editing, supervision, project administration. **Cathy Allen**: conceptualisation, methodology, writing – review and editing, supervision. All authors agree to be accountable for all aspects of the work.

## Funding

The authors have nothing to report.

## Conflicts of Interest

The authors declare no conflicts of interest.
